# Portable Raman spectroscopy combined with machine learning for rapid and label-free recognition of five pathogenic bacteria

**DOI:** 10.3389/fmicb.2026.1872435

**Published:** 2026-08-06

**Authors:** Shisheng Cao, Ran Pang, Yongqiang Chen, Langdi Zhong, Xiaoxi Dong, Hongxiao Li, Huijuan Yin

**Affiliations:** 1 Institute of Biomedical Engineering, Chinese Academy of Medical Sciences and Peking Union Medical College, Tianjin, China; 2 Department of Orthopedics, General Hospital of Tianjin Medical University, Tianjin, China

**Keywords:** antimicrobial resistance, bacterial identification, machine learning, portable diagnostics, Raman spectroscopy

## Abstract

**Introduction:**

Antimicrobial resistance (AMR) continues to rise globally, highlighting the need for rapid, label-free, and cost-effective bacterial identification methods. In this proof-of-concept study, portable Raman spectroscopy combined with machine learning was used to identify five clinically relevant bacterial species: *Escherichia coli, Pseudomonas aeruginosa, Staphylococcus aureus, Porphyromonas gingivalis*, and *Streptococcus mutans*.

**Methods:**

Raman spectra were acquired from cultured, washed, PBS-resuspended, and OD-standardized bacterial suspensions. After SNIP baseline correction, binary and five-class classification models were constructed using Auto-Sklearn with eight algorithms: ADB, ET, GB, LDA, SVM, MLP, PA, and QDA. Model performance was evaluated using accuracy, precision, recall, *F*1-score, MCC, and ROC-AUC.

**Results:**

Pairwise binary classification showed variable performance among bacterial pairs. The best result was obtained for *P. gingivalis* versus *S. aureus*, with a testing accuracy of 98.3%, precision of 0.984, recall of 0.983, *F*1-score of 0.983, MCC of 0.967, and ROC-AUC of 1.000. Five-class classification was more limited, with LDA achieving the highest testing accuracy of 60.1%, MCC of 0.506, and ROC-AUC of 0.867.

**Discussion:**

These findings support the feasibility of portable Raman spectroscopy combined with machine learning for bacterial recognition under standardized sample conditions.

## 1 Introduction

Antimicrobial resistance (AMR) has emerged as one of the most critical global public health challenges (Okeke et al., 2024). The increasing prevalence of antibiotic-resistant bacterial strains has made infectious diseases more difficult to treat, resulting in higher mortality rates and substantial healthcare costs (de la Fuente-Nunez et al., 2023). Although bacterial resistance can naturally evolve, excessive and inappropriate use of antibiotics in clinical practice has greatly accelerated its emergence and dissemination (Sati et al., 2025; SeyedAlinaghi et al., 2025). Currently, definitive bacterial diagnosis in clinical settings still relies primarily on conventional culture and biochemical identification methods, which require several hours or even days to complete and often fail to provide timely guidance for targeted antibiotic therapy (Ahmadi et al., 2025). In the absence of rapid and accurate pathogen detection tools, clinicians frequently resort to empirical use of broad-spectrum antibiotics, further exacerbating antibiotic misuse and resistance (Rentschler et al., 2021). This issue is particularly critical in resource-limited or point-of-care settings, where access to well-equipped laboratories and skilled personnel is limited, highlighting the urgent need for rapid, reliable, and field-deployable diagnostic approaches for bacterial identification (Sharma et al., 2023).

Consequently, developing rapid, accurate, and accessible bacterial identification methods has become an important goal for precision anti-infective therapy and for controlling the spread of antimicrobial resistance (Hamada et al., 2021). Among emerging diagnostic technologies, Raman spectroscopy has attracted increasing attention due to its non-destructive, label-free nature and its ability to provide molecular vibrational “fingerprint” information (X. Li et al., 2026; Locke et al., 2020; Mushenkov et al., 2025). By analyzing the molecular composition and structural differences reflected in bacterial Raman spectra, rapid bacterial identification can be achieved without complex sample preparation—a feature that makes Raman spectroscopy particularly advantageous for on-site testing in resource-limited or point-of-care environments (Usman et al., 2023).

Currently, the most widely used Raman-based techniques include confocal Raman microscopy and surface-enhanced Raman spectroscopy (SERS) (Hassanain et al., 2024; Y. Wang et al., 2025). Confocal Raman microscopy enables single-cell spectral acquisition with high spatial resolution and excellent quantitative capability; however, its complex operation, slow scanning speed, and high instrumentation costs hinder its clinical deployment (J. Zhang et al., 2022). In contrast, SERS amplifies Raman signals through localized surface plasmon enhancement on metallic nanostructures, significantly improving detection sensitivity and enabling low-concentration or single-cell detection (Dina et al., 2023). Nevertheless, the reproducibility and stability of SERS signals remain key limitations for clinical translation, as small variations in substrate fabrication can lead to signal fluctuations and affect detection reliability (Y. Li et al., 2022).

Overall, while Raman spectroscopy shows great promise for rapid bacterial identification, existing systems are constrained by detection speed, signal stability, and instrument portability (Tan et al., 2024). Furthermore, bacterial Raman spectra are inherently high-dimensional and feature-overlapping, making it difficult for conventional statistical methods to achieve accurate classification (Bi et al., 2024). With the rapid development of artificial intelligence, machine learning algorithms have emerged as powerful tools for spectral data analysis and pattern recognition, enabling automated extraction of discriminative spectral features and improving bacterial classification accuracy (Liu et al., 2024).

**Figure 1:**
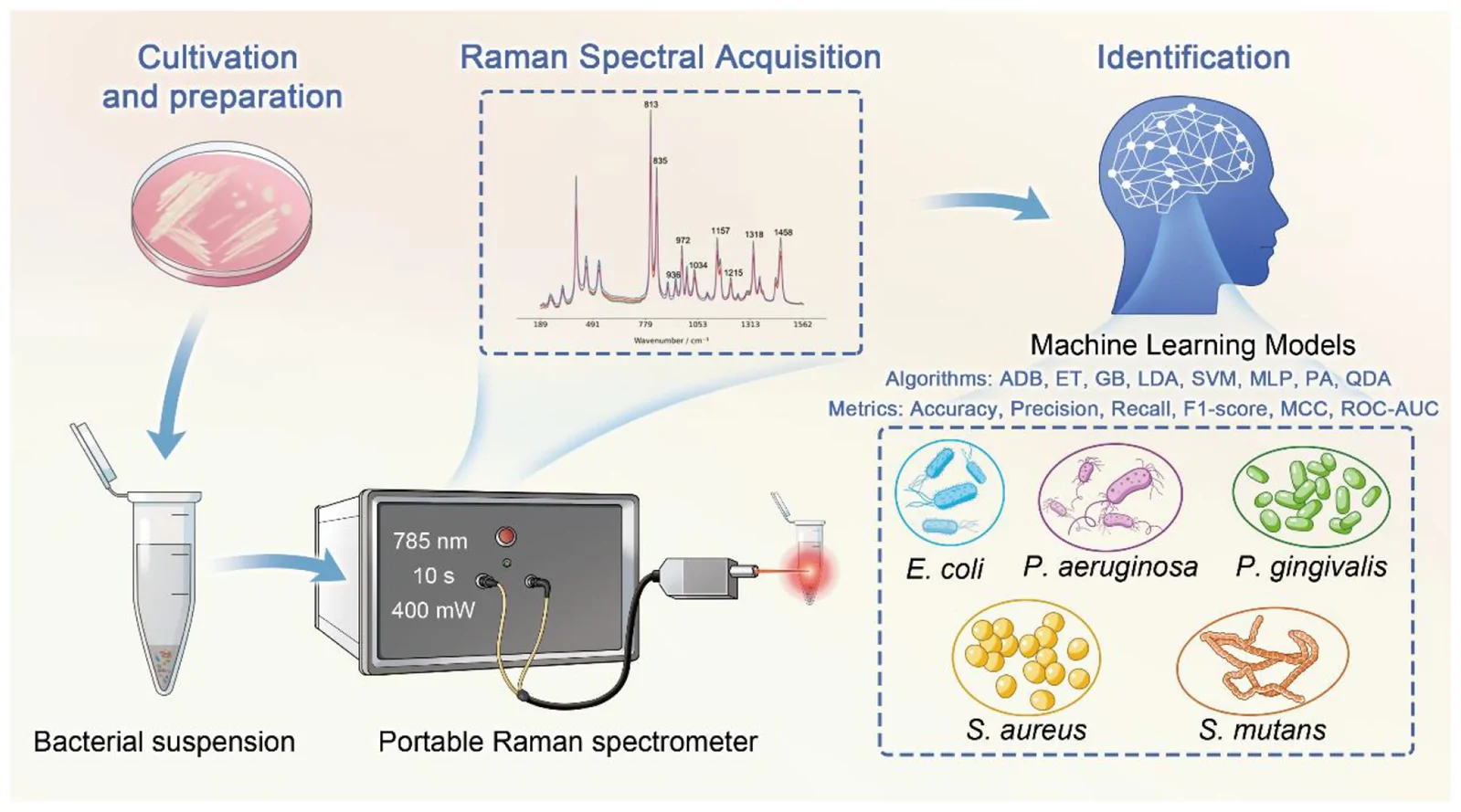
Workflow of portable Raman spectroscopy combined with machine learning for bacterial recognition. Raman spectra were acquired from prepared bacterial suspensions using a portable Raman spectrometer and analyzed using eight machine learning algorithms: ADB, ET, GB, LDA, SVM, MLP, PA, and QDA. Model performance was evaluated using accuracy, precision, recall, F1-score, MCC, and ROC-AUC.

Based on these considerations, this study proposes a portable Raman spectroscopy–machine learning workflow as a label-free optical recognition strategy for the recognition of prepared bacterial suspensions (Figure 1). Raman spectra were collected from five clinically relevant bacterial species — *Escherichia coli, Pseudomonas aeruginosa, Staphylococcus aureus, Porphyromonas gingivalis*, and *Streptococcus mutans*—which represent both Gram-negative and Gram-positive bacteria, include aerobic and anaerobic organisms, and cover pathogens associated with systemic and oral infections. Eight machine learning models were then trained and optimized to evaluate both pairwise and five-class discrimination. The main objective of this study was to assess whether portable Raman fingerprints, combined with machine learning, could provide a rapid and label-free recognition workflow for prepared bacterial suspensions. This approach may support the future development of accessible pathogen identification tools under simplified or resource-limited conditions, although further validation using clinical specimens and external datasets will be required before practical diagnostic application.

## 2 Materials and methods

### 2.1 Bacterial culture and pretreatment

Five clinically significant bacterial species were included in this study: *Escherichia coli* (*E. coli*), *Pseudomonas aeruginosa* (*P. aeruginosa*), *Staphylococcus aureus* (*S. aureus*), *Porphyromonas gingivalis* (*P. gingivalis*), and *Streptococcus mutans* (*S. mutans*), For each bacterial species, one reference strain was used, and 50 independently prepared culture-derived bacterial samples were analyzed. In this study, each physical sample refers to one independently prepared bacterial suspension or culture-derived sample, rather than an independent clinical or environmental isolate.

*E. coli, P. aeruginosa*, and *S. aureus* were streaked on LB agar plates and incubated aerobically at 37 °C for 24 h. A single colony from each plate was then inoculated into 5 mL of LB broth and cultured under the same conditions for another 24 h to obtain pure bacterial suspensions. *P. gingivalis* and *S. mutans* were cultured in BHI broth and CDC agar plates, respectively, under anaerobic conditions at 37 °C.

After incubation, the bacterial suspensions were centrifuged at 5,000 rpm for 10 min, and the supernatant was discarded. The pellets were washed three times with 5 mL of phosphate-buffered saline (PBS) to remove any residual medium components that could affect Raman measurements. The final bacterial suspensions were adjusted to an optical density of OD_630_ = 1.0 in PBS and stored at 4 °C until use (Thomsen et al., 2022).

### 2.2 Raman spectral acquisition

One milliliter of bacterial suspension with identical optical density (OD_630_ = 1.0) was transferred into a sterile 1.5 mL disposable microcentrifuge tube, which served as the sample carrier for Raman measurement. Before spectral acquisition, each suspension was gently mixed to reduce sedimentation-related variation. Raman spectra were collected directly from the bacterial suspension in the microcentrifuge tube using a portable Raman spectrometer (ATR3110-785, Optosky Co., Ltd., China). The instrument employed a 785 nm excitation laser with a pixel resolution of 6 cm^−1^ and a spectral range of 200 cm^−1^−2,600 cm^−1^. All measurements were performed at room temperature using fixed acquisition settings, with a laser power of 400 mW and an integration time of 10 s. Each sample was measured 10 times as technical replicate spectra under the same acquisition conditions, resulting in approximately 2,500 Raman spectra in total. Each spectrum contained 2,048 data points covering the 200 cm^−1^−2,600 cm^−1^ wavenumber range.

### 2.3 Spectral preprocessing

Raw Raman spectra were exported from the portable Raman spectrometer and processed using a Python-based preprocessing pipeline before machine-learning analysis. The pipeline was implemented using the pandas, numpy, and scipy libraries.

No additional mathematical smoothing, such as Savitzky–Golay filtering, was applied after spectral acquisition. Manual cosmic ray removal was also not performed during post-acquisition processing, in order to avoid introducing additional user-dependent preprocessing effects. The raw spectra were first subjected to baseline correction using a custom implementation of the statistics-sensitive non-linear iterative peak-clipping (SNIP) algorithm. Before iterative clipping, the spectral intensity values were transformed using the log–log–square-root transformation: $y^{\prime }=\ln\left(\ln\left(\sqrt{y+1}+1\right)+1\right)$ where *y* represents the original spectral intensity. The SNIP clipping window was empirically adjusted within the range of 50–200 points to estimate the broad fluorescence background while preserving relatively sharp Raman peaks. To maintain consistency across spectra, a union baseline strategy was applied, in which a global average baseline was estimated and subtracted from individual spectra (Han et al., 2024; Yan, 2025).

After baseline correction, an outlier exclusion step was performed to remove spectra with severe acquisition artifacts or gross signal deviations. For each spectrum, the total absolute difference from the overall mean spectrum was calculated. Spectra showing extreme deviation from the global spectral distribution were identified as outliers and excluded before downstream machine-learning analysis.

No fixed manual normalization method, such as Standard Normal Variate (SNV), vector normalization, or min–max scaling, was applied during the initial preprocessing stage. To reduce sample-level intensity variation before spectral acquisition, all bacterial suspensions were standardized to OD_630_ = 1.0, and Raman spectra were collected under fixed instrumental parameters. During model construction, scaling and preprocessing operations available within the Auto-Sklearn framework were allowed to be selected automatically during hyperparameter optimization, depending on the requirements of each algorithm. The resulting preprocessed spectra were then used as input features for binary and five-class classification models.

### 2.4 Construction and training of binary classification models

Baseline correction was applied to the raw Raman spectra prior to model training to improve spectral quality and signal-to-noise ratio. Baselines were estimated and removed using the statistics-sensitive non-linear iterative peak-clipping (SNIP) algorithm. To evaluate the discrimination capability between any two bacterial species, multiple binary classification models were constructed using the automated machine learning framework Auto-Sklearn. It is worth noting that explicit manual spectral normalization was bypassed prior to modeling; instead, optimal data scaling and normalization strategies were dynamically selected and applied by the Auto-sklearn framework during its automated hyperparameter optimization phase. Since five bacterial classes were included, a total of 10 binary combinations were generated, resulting in 10 classification models.

Machine-learning model construction was performed using Auto-Sklearn version 0.13.0, an automated machine-learning framework for model selection and hyperparameter optimization. The software documentation and release information are available from the official Auto-Sklearn documentation and PyPI repository: https://automl.github.io/auto-sklearn/; https://pypi.org/project/auto-sklearn/. To evaluate the discrimination capability between bacterial species, both pairwise binary classification and five-class classification tasks were conducted. Since five bacterial species were included, all possible pairwise combinations were generated, resulting in 10 binary classification tasks. In addition, a five-class model was constructed to simultaneously distinguish all five bacterial species.

Eight machine-learning algorithms were evaluated: Adaptive Boosting (ADB), Extremely Randomized Trees (ET), Gradient Boosting (GB), Linear Discriminant Analysis (LDA), Support Vector Machine with LibSVM SVC (SVM), Multilayer Perceptron (MLP), Passive Aggressive classifier (PA), and Quadratic Discriminant Analysis (QDA). For each training run, only one specified learner was included through the include_estimators parameter, allowing direct comparison among individual algorithms. Hyperparameter optimization was performed automatically within the default Auto-Sklearn search space for each included learner. Unless otherwise specified, default Auto-Sklearn settings were used.

For model optimization, the time budget was set to 1,800 s for each pairwise binary classification task and 3,600 s for the five-class classification task. The number of parallel jobs was set to 30, and the memory limit was set to 32,000 MB. The ensemble size was set to 1; therefore, ensemble learning was disabled, and only the best single model was retained. Cross-validation was used as the resampling strategy, with the number of folds set to three. The main Auto–Sklearn configuration and model-training parameters are summarized in Supplementary Table S1. Data were split at the level of the physical sample (one culture tube treated as one sample), with all replicate spectra from a given sample kept in the same partition. In this study, one physical sample corresponded to one independently prepared culture-derived bacterial suspension, and each physical sample generated 10 technical replicate Raman spectra. The dataset was divided into a training + validation portion (75%) and a separate test set (25%). Within the training + validation portion, group-wise splits produced the training and validation subsets: the training subset was used for model fitting, and the validation subset for monitoring performance and selecting hyperparameters. During these group-wise splits, all technical replicate spectra from the same physical sample were kept within the same subset to maintain sample-level independence. The test set was not used during model development and was reserved for final evaluation. The dataset structure and sample-level partitioning are summarized in Supplementary Table S2.

Within the training/validation portion described above, model training and hyperparameter optimization were performed using three-fold group-wise cross-validation. The group-wise setting was used to maintain sample-level independence during cross-validation. The mean validation performance across the three folds was used to guide automated hyperparameter optimization and identify the best-performing model configuration. After model selection, the optimized model was evaluated on the independent test set, which was not used during model training, validation, or hyperparameter tuning.

Model performance was assessed using accuracy, precision, recall, *F*1-score, Matthews correlation coefficient (MCC), and receiver operating characteristic area under the curve (ROC-AUC). For binary classification, class-wise precision, recall, and *F*1-score were calculated for each bacterial species, and macro-averaged precision, recall, and *F*1-score were reported to summarize overall performance across both classes. MCC was used as an additional balanced metric, and ROC-AUC was calculated to evaluate the discriminative ability of each model. Support was defined as the number of test spectra belonging to each class (Chicco and Jurman, 2023; Rainio et al., 2024).

### 2.5 Construction and training of the five-class classification model

In addition to pairwise binary classification, a five-class classification model was constructed to evaluate the simultaneous discrimination of all five bacterial species, including *E. coli, P. aeruginosa, S. aureus, P. gingivalis*, and *S. mutans*. The same preprocessed Raman spectra, sample-level data partitioning strategy, and Auto-Sklearn framework described above were used for the five-class task. The five bacterial species were treated as independent output classes.

For the five-class classification task, the Auto-Sklearn time budget was set to 3,600 s. Other major settings, including candidate algorithms, cross-validation strategy, number of parallel jobs, memory limit, and ensemble size, were kept consistent with the binary classification models. The optimized models were evaluated on the independent test set. Model performance was assessed using accuracy, macro-averaged precision, macro-averaged recall, macro-averaged *F*1-score, MCC, and ROC-AUC. Normalized confusion matrices and class-wise precision, recall, *F*1-score, and support were further generated to analyze species-level classification performance and inter-class misclassification patterns.

## 3 Results

### 3.1 Raman spectral feature analysis

**Figure 2:**
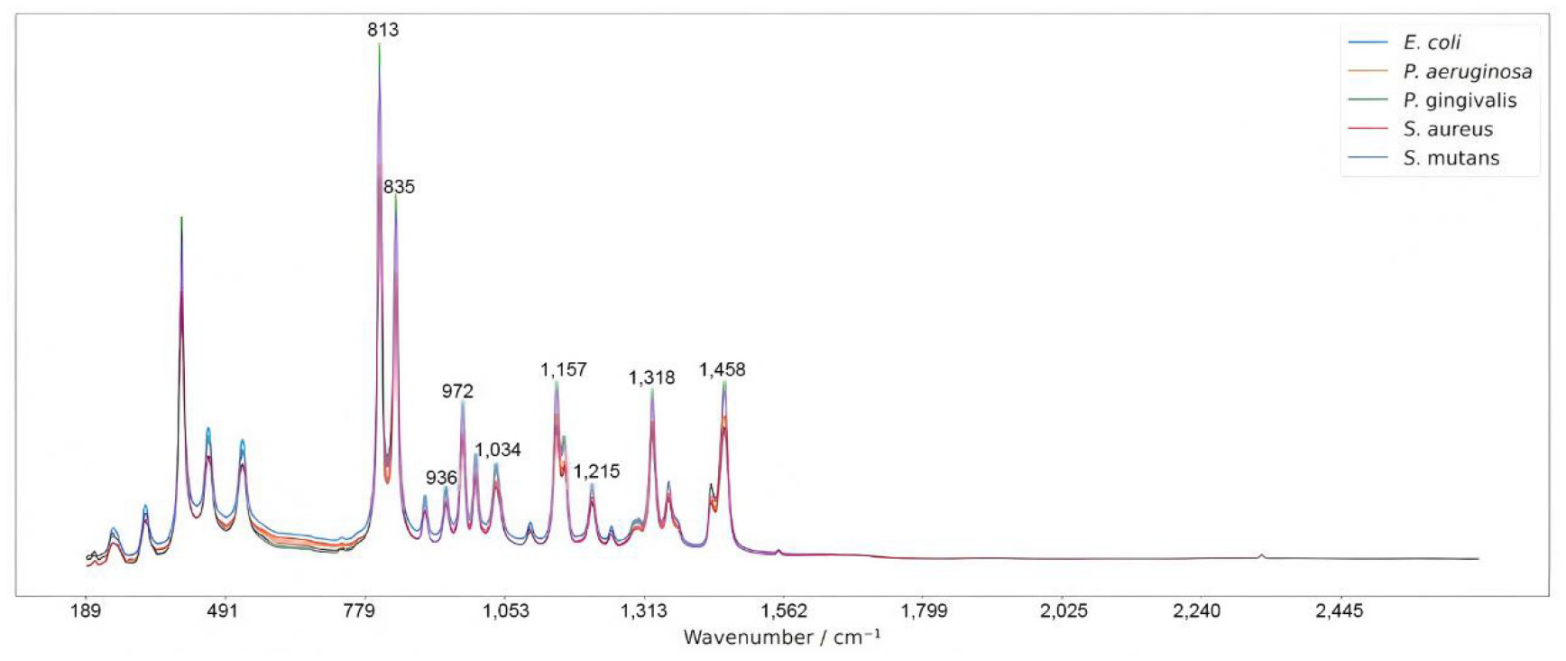
Representative Raman spectra of five common pathogenic bacteria: *Escherichia coli, Pseudomonas aeruginosa, Porphyromonas gingivalis, Staphylococcus aureus*, and *Streptococcus mutans*. Distinct Raman peaks were observed at 813, 835, 936, 972, 1,034, 1,157, 1,215, 1,318, and 1,458 cm^−1^, corresponding to molecular vibrations of proteins, nucleic acids, and lipids. The spectra demonstrate overall similarity in the major spectral profiles, yet subtle variations in peak intensity and position reveal compositional differences among bacterial species.

The representative Raman spectra of the five bacterial species are shown in Figure 2. Overall, the spectra exhibited similar global profiles within the range of 200 cm^−1^−2,600 cm^−1^, suggesting that the major biochemical components of these bacterial cells generated broadly comparable Raman spectral patterns. However, differences in relative peak intensity and local spectral shape were observed at several characteristic bands, including 813 cm^−1^, 835 cm^−1^, 936 cm^−1^, 972 cm^−1^, 1,034 cm^−1^, 1,157 cm^−1^, 1,215 cm^−1^, 1,318 cm^−1^, and 1,458 cm^−1^.

The bands around 813 cm^−1^ and 835 cm^−1^ were assigned mainly to nucleic-acid backbone vibrations and tyrosine-related Fermi resonance, respectively. The 936 cm^−1^ band may be associated with protein backbone C–C stretching, while the 972 cm^−1^ band is related to lipid or polysaccharide skeletal vibrations. The band at 1,034 cm^−1^ contains contributions from aromatic amino acid residues, such as phenylalanine/proline, as well as carbohydrate-related C–O/C–C stretching. The 1,157 cm^−1^ band may be linked to C–C stretching of carotenoid-like polyene chains, whereas the 1,215 cm^−1^ and 1,318 cm^−1^ bands are mainly associated with amide III vibrations and protein-related CH_2_/CH_3_ deformation. The 1,458 cm^−1^ band corresponds to CH_2_/CH_3_ deformation in lipids and proteins (Efrima and Zeiri, 2008; Jung et al., 2014; Mosier-Boss, 2017). These peak assignments are summarized in Table 1.

**Table 1:** Putative Raman peak assignments in bacterial spectra.

Raman shift (cm^−1^)	Putative band assignment	Putative biochemical contribution
813	O–P–O/C–O–P–O–C backbone modes	RNA/DNA phosphodiester backbone (Movasaghi et al., 2007)
835	Tyr Fermi resonance (low-wavenumber component ~830–850); minor backbone contribution possible	Tyrosine (protein); possible nucleic-acid backbone (Movasaghi et al., 2007)
936	C–C skeletal stretch (α-helix); minor polysaccharide C–C	Protein backbone (α-helical content); polysaccharides (Movasaghi et al., 2007)
972	C–C skeletal stretch (lipids/polysaccharides); weak phosphate-related modes possible	Lipids and polysaccharides (Jehlicka et al., 2014)
1,034	Aromatic residues (Phe/Pro) C–H in-plane deformation; C–O/C–C (glycans, LPS)	Proteins (aromatic aa) and carbohydrates/LPS (Cheng et al., 2005)
1,157	Polyene C–C stretch of carotenoids	Carotenoid pigments (cell envelope) (P. Zhang et al., 2024)
1,215	Aromatic residues/amide-III contributions (C–C/C–N); ${\text{PO}}_{2}^{-}$ asymmetric stretch typically at 1,240–1,260	Proteins (Tyr/Phe/Trp; amide-III); minor nucleic-acid phosphate (P. Zhang et al., 2024)
1,318	Amide-III (random/β) + CH_2_/CH_3_ deformation; possible nucleic-acid contributions	Proteins (backbone + side chains); minor nucleic acids (Dincturk, 2024)
1,458	CH_2_/CH_3_ scissoring deformation	Lipids and proteins (Dincturk, 2024)

Although these Raman bands are not unique to a single bacterial species, variations in their relative intensities and combined spectral patterns reflect differences in cellular biochemical composition, including proteins, nucleic acids, lipids, and polysaccharides. These subtle spectral differences provide informative features for subsequent machine-learning-based classification. At the same time, the overall similarity and partial overlap of bacterial Raman spectra may explain why binary classification was generally more accurate than five-class discrimination.

### 3.2 Performance evaluation of binary classification models

**Figure 3:**
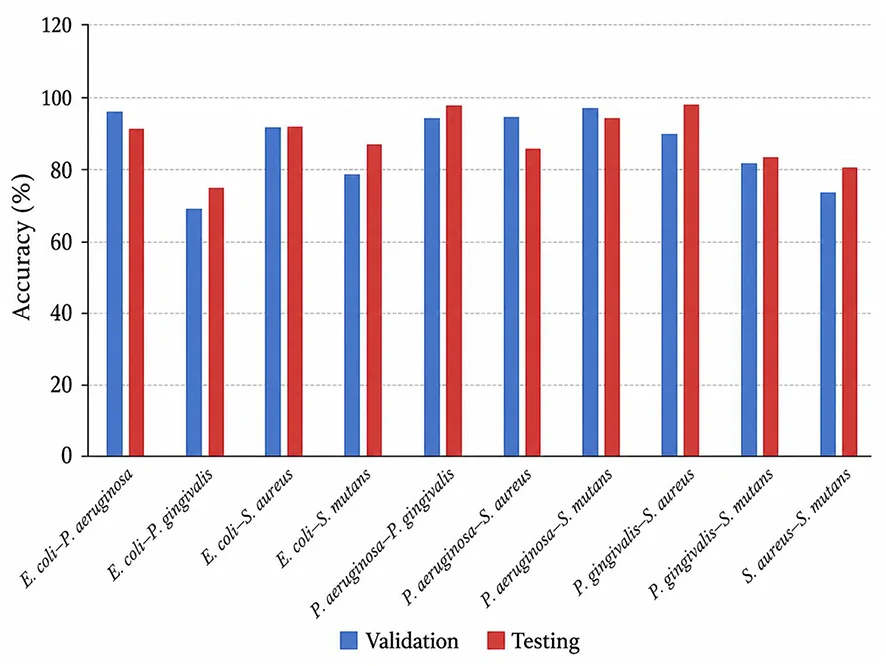
Validation and testing accuracies of binary classification models for pairwise bacterial discrimination based on Raman spectra. Ten model groups were constructed from all possible pairwise combinations of the five bacterial species (*E. coli, P. aeruginosa, P. gingivalis, S. aureus*, and *S. mutans*). Each bar represents the classification accuracy on the validation set (blue) and the testing set (red).

To evaluate the pairwise discriminability of the five bacterial species, binary classification models were constructed for all possible species pairs. In total, ten pairwise models were generated and assessed using validation and testing accuracy (Figure 3). Overall, the validation and testing accuracies showed similar trends across most bacterial pairs, suggesting no obvious evidence of overfitting under the current group-wise data-splitting strategy. However, classification performance varied across different bacterial pairs, indicating that Raman spectral separability differed among species. The highest testing accuracy was observed for the *P. gingivalis*–*S. aureus* pair (98.3%), whereas the lowest testing accuracy was obtained for *E. coli*–*P. gingivalis* (74.5%). These differences may be associated with variations in biochemical composition and spectral overlap between bacterial species.

To further summarize the performance of the binary classification models, macro-averaged precision, macro-averaged recall, macro-averaged *F*1-score, accuracy, and support were calculated for each bacterial pair (Table 2). These results were generally consistent with the accuracy trends shown in Figure 3. Most binary models achieved testing accuracy values above 80.0%, indicating relatively good pairwise discrimination performance. Among all bacterial pairs, the best performance was observed for *P. gingivalis* versus *S. aureus*, with an accuracy of 98.3%, macro-averaged precision of 0.984, macro-averaged recall of 0.983, macro-averaged *F*1-score of 0.983, MCC of 0.967, and ROC-AUC of 1.000. High accuracy was also obtained for *P. aeruginosa* versus *P. gingivalis* (97.5%) and *P. aeruginosa* versus *S. mutans* (93.8%). In contrast, the lowest accuracy was observed for *E. coli* versus *P. gingivalis* (74.5%), with an MCC of 0.497 and ROC-AUC of 0.821, suggesting greater spectral overlap or weaker class separation for this bacterial pair. Overall, the macro-averaged metrics, MCC, and ROC-AUC values support the feasibility of pairwise bacterial discrimination while also highlighting clear differences in classification difficulty among bacterial pairs. The full class-wise precision, recall, *F*1-score, and support values for each binary model are provided in Table S3.

**Table 2:** Performance metrics of binary classification models.

Bacterial pair	Macro precision	Macro recall	Macro *F*1-score	Accuracy (%)	MCC	ROC-AUC	Support
*E. coli* vs *P. aeruginosa*	0.917	0.908	0.907	90.8	0.825	0.92	239
*E. coli* vs *P. gingivalis*	0.753	0.744	0.743	74.5	0.497	0.821	239
*E. coli* vs *S. aureus*	0.916	0.912	0.912	91.2	0.828	0.957	239
*E. coli* vs *S. mutans*	0.862	0.862	0.862	86.2	0.724	0.947	239
*P. aeruginosa* vs *P. gingivalis*	0.975	0.975	0.975	97.5	0.95	0.995	240
*P. aeruginosa* vs *S. aureus*	0.873	0.85	0.848	85	0.723	0.82	240
*P. aeruginosa* vs *S. mutans*	0.938	0.938	0.937	93.8	0.875	0.988	240
*P. gingivalis* vs *S. aureus*	0.984	0.983	0.983	98.3	0.967	1	240
*P. gingivalis* vs *S. mutans*	0.85	0.825	0.822	82.5	0.674	0.933	240
*S. aureus* vs *S. mutans*	0.809	0.808	0.808	80.8	0.617	0.893	240

To further examine the discriminative characteristics of individual bacterial species across the dataset, a one-vs-rest analysis was subsequently performed. This approach assessed how well each species could be distinguished from the remaining four species and provided additional information on species-level spectral separability.

**Figure 4:**
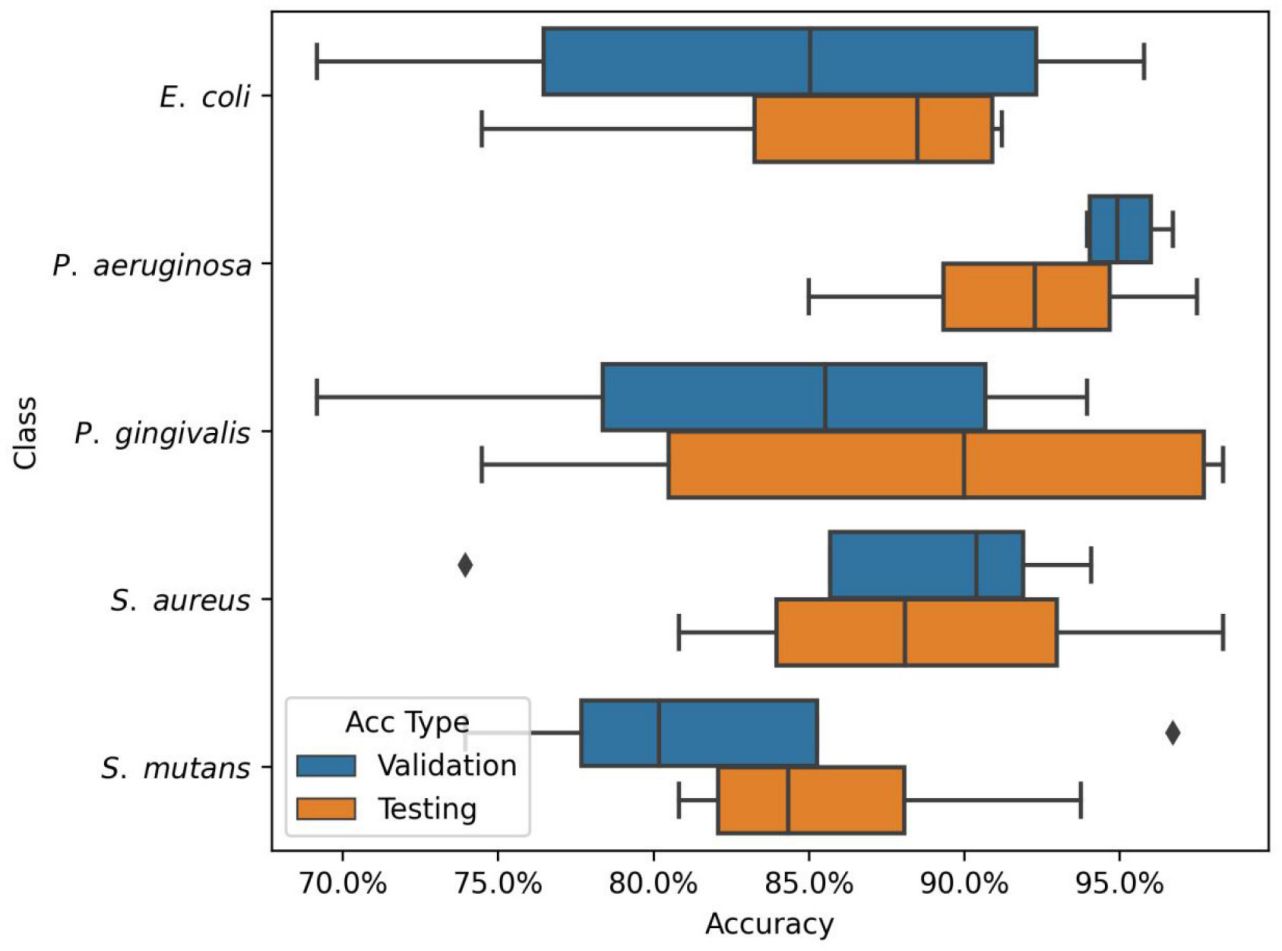
Validation and testing accuracy distributions of one-vs-rest classification models for the five bacterial species based on Raman spectra. For each species, the boxplot summarizes the accuracies obtained from binary comparisons between that species and each of the remaining four species. Higher accuracy suggests greater separability under the current model, while shorter box lengths indicate more consistent classification performance across comparisons.

To further assess species-level separability, the accuracy distributions of one-vs-rest classification models were analyzed (Figure 4). For each bacterial species, the boxplot summarizes the accuracies obtained when that species was classified against each of the remaining four species. Higher accuracy values suggest greater separability under the current Raman–machine learning framework, whereas shorter box lengths indicate more consistent classification performance across pairwise comparisons.

Overall, *E. coli* showed relatively high and consistent accuracy in both validation and testing sets, suggesting that it was more readily distinguished from the other species in this dataset. *P. gingivalis* and *S. aureus* exhibited intermediate levels of separability, while *S. mutans* showed lower and more variable accuracy, indicating that its Raman spectral features may overlap more substantially with those of other bacteria. These findings further support that classification difficulty differs among bacterial species and depends on the degree of spectral similarity between them.

**Figure 5:**
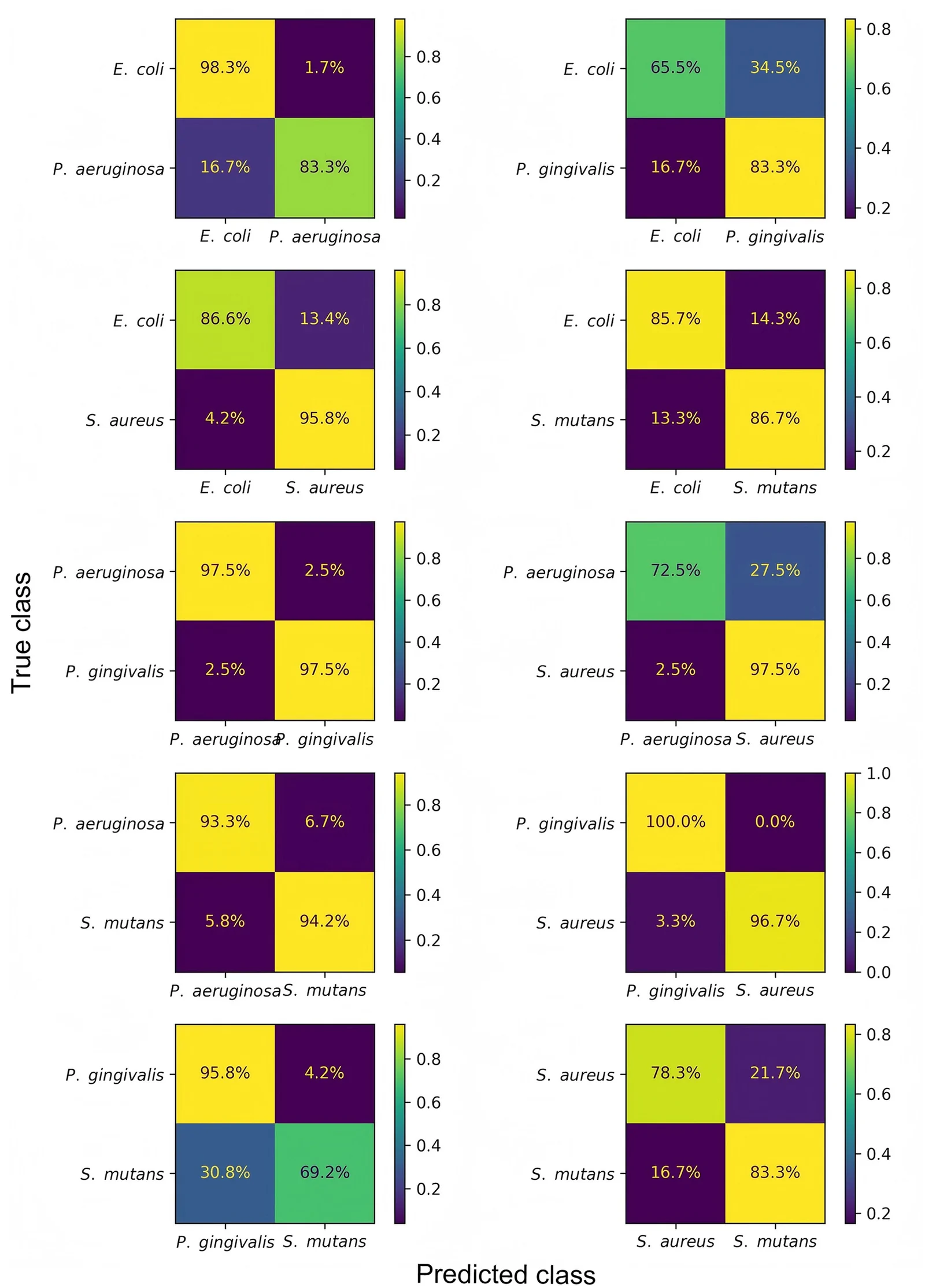
Normalized confusion matrices of binary classification models for pairwise bacterial discrimination based on Raman spectra. Each submatrix represents the classification performance for one bacterial pair. Rows indicate true labels, and columns indicate predicted labels. Values in each cell represent the proportion of spectra assigned to each predicted class. Diagonal values indicate correct classification rates, whereas off-diagonal values indicate misclassification proportions.

To further examine the classification behavior of the binary models, confusion matrix analysis was performed for all pairwise bacterial combinations (Figure 5). Overall, most models showed relatively high diagonal values, indicating that the majority of test spectra were correctly assigned. However, the confusion matrices also revealed pair-dependent differences and asymmetric misclassification patterns.

The strongest discrimination was observed for the *P. gingivalis*–*S. aureus* pair, with diagonal values of 1.000 and 0.967 for *P. gingivalis* and *S. aureus*, respectively. The *P. aeruginosa*–*P. gingivalis* pair also showed high and balanced classification performance, with both diagonal values reaching 0.975. In contrast, some bacterial pairs exhibited more pronounced misclassification. For example, in the *E. coli*–*P. gingivalis* model, 34.5% of *E. coli* spectra were misclassified as *P. gingivalis*. Similarly, in the *P. gingivalis*–*S. mutans* model, 30.8% of *S. mutans* spectra were misclassified as *P. gingivalis*. These results suggest that specific bacterial pairs may share more overlapping Raman spectral features, which can reduce classification performance.

Taken together, the confusion matrix results support the feasibility of using portable Raman spectroscopy combined with machine learning for pairwise bacterial discrimination. At the same time, the observed misclassification patterns indicate that spectral similarity between certain species remains a limitation, particularly for extending the approach to more complex multi-class classification tasks.

### 3.3 Performance of the five-class classification model

**Figure 6:**
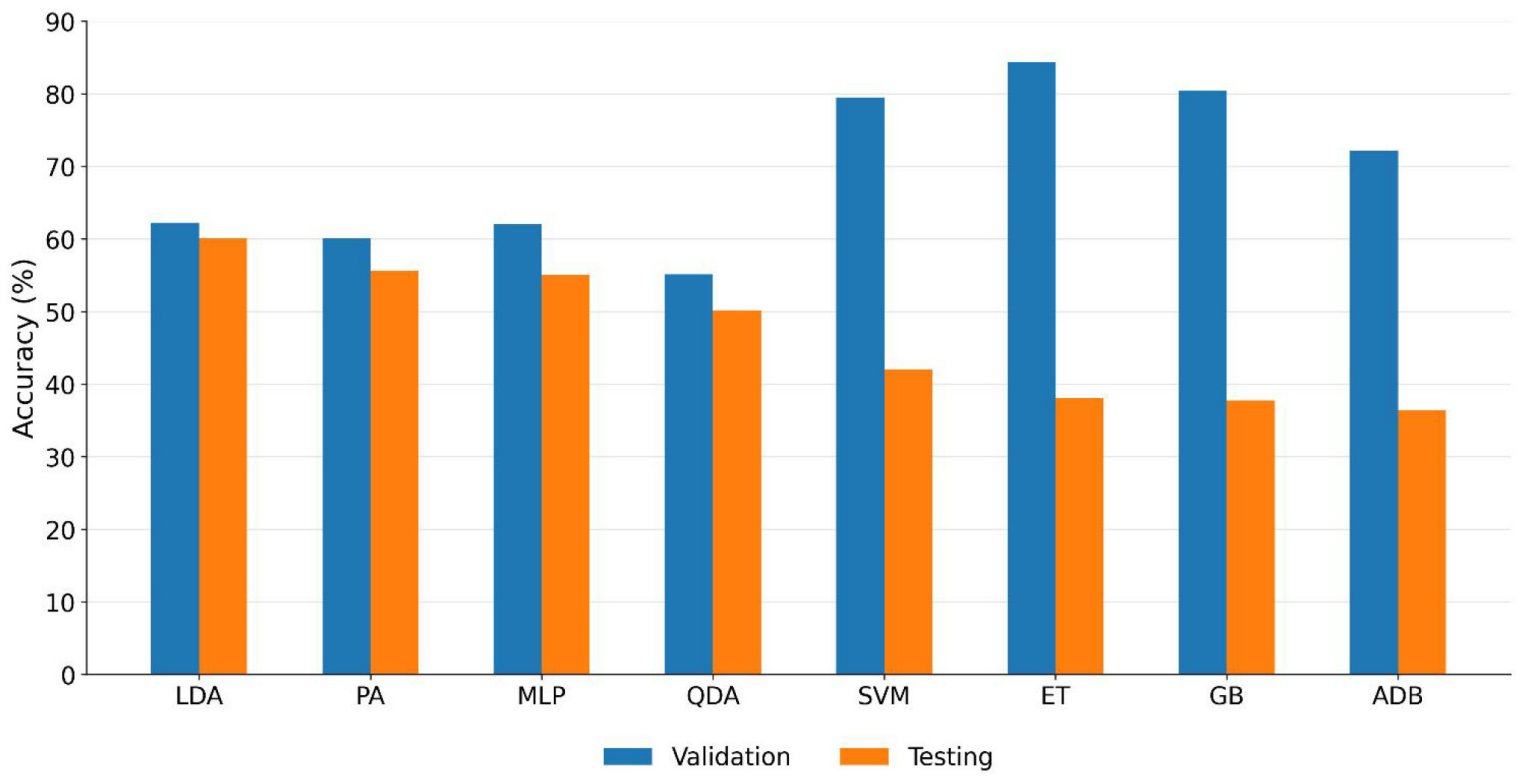
Comparison of validation and testing accuracies for the five-class classification task across eight machine learning algorithms. Blue bars represent validation accuracy, and red bars represent testing accuracy. The relatively small validation–testing gaps observed for LDA, PA, MLP, and QDA suggest more stable performance, whereas larger gaps for SVM, ET, GB, and ADB indicate limited generali zation to the test set.

After evaluating pairwise bacterial discrimination, we further assessed whether the Raman–machine learning workflow could be extended to simultaneous five-species classification. Eight machine learning algorithms were compared for the five-class classification task, and their validation and testing accuracies are shown in Figure 6.

Clear differences were observed among the algorithms. LDA, PA, MLP, and QDA showed relatively small gaps between validation and testing accuracies, suggesting no obvious evidence of overfitting under the current group-wise validation strategy. Among these algorithms, LDA achieved validation and testing accuracies of 62.2% and 60.1%, respectively, while PA, MLP, and QDA showed testing accuracies between 50.1% and 55.6%. The overall MCC and ROC-AUC values further supported this trend (Table S4). LDA achieved the highest MCC of 0.506 and ROC-AUC of 0.867, followed by PA (MCC = 0.452; ROC-AUC = 0.841), MLP (MCC = 0.439; ROC-AUC = 0.844), and QDA (MCC = 0.377; ROC-AUC = 0.774).

In contrast, SVM, ET, GB, and ADB showed substantially higher validation accuracies than testing accuracies, indicating limited generalization to the independent test set. Although ET and GB achieved validation accuracies above 80.0%, their testing accuracies were below 40.0%, suggesting that these models were more strongly affected by overfitting in the five-class task. Consistently, ADB, ET, and GB showed relatively low MCC values of 0.207, 0.228, and 0.223, respectively, and ROC-AUC values below 0.690 (Table S4).

**Figure 7:**
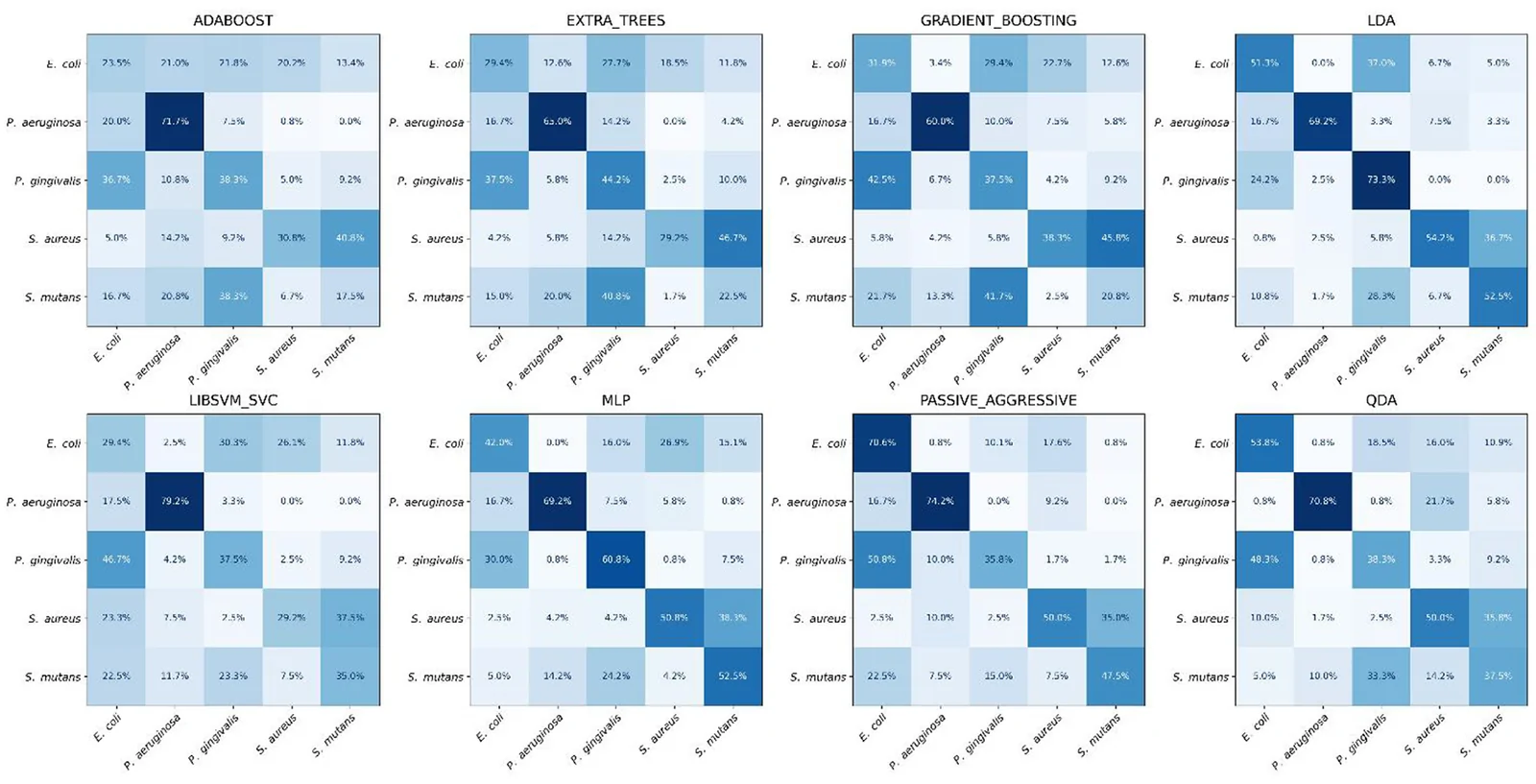
Normalized confusion matrices for the five-class bacterial classification task across eight machine learning algorithms. Confusion matrices are shown for Adaptive Boosting (ADABOOST), Extremely Randomized Trees (EXTRA_TREES), Gradient Boosting (GRADIENT_BOOSTING), Linear Discriminant Analysis (LDA), Support Vector Machine with LibSVM SVC (LIBSVM_SVC), Multilayer Perceptron (MLP), Passive Aggressive classifier (PASSIVE_AGGRESSIVE), and Quadratic Discriminant Analysis (QDA). Rows indicate the true bacterial species, and columns indicate the predicted bacterial species. Values in each cell represent the percentage of test spectra assigned to each predicted class. Diagonal values indicate correct classification, whereas off-diagonal values indicate misclassification between bacterial species.

The normalized confusion matrices provided further insight into the species-level classification patterns (Figure 7). For the LDA model, the correct classification rates for *E. coli, P. aeruginosa, P. gingivalis, S. aureus*, and *S. mutans* were 51.3%, 69.2%, 73.3%, 54.2%, and 52.5%, respectively. Class-wise metrics also showed that P. aeruginosa was relatively well recognized by several algorithms, with *F*1-scores of 0.787 for LDA, 0.772 for SVM, 0.735 for MLP, 0.733 for PA, and 0.769 for QDA (Table S5). In contrast, the remaining species showed more variable performance across algorithms, indicating algorithm-dependent inter-class confusion in the five-class task.

Overall, LDA, PA, MLP, and QDA provided more stable performance among the tested algorithms, with testing accuracies above the theoretical random baseline of 20%. However, the five-class classification performance remained moderate, indicating that simultaneous discrimination of multiple bacterial species is still challenging under the current dataset and portable Raman acquisition conditions. Together, the accuracy, confusion matrix, class-wise metric, MCC, and ROC-AUC results indicate that portable Raman spectroscopy combined with machine learning can capture useful discriminatory information for five-class bacterial recognition, but its current multiclass performance remains limited compared with pairwise binary classification.

## 4 Discussion

Traditional bacterial identification methods mainly include morphological and molecular biological approaches (B. Wang et al., 2023). Gram staining, as the most commonly used preliminary screening method, is simple but highly operator-dependent and limited in accuracy (Hussain et al., 2024). Molecular-based methods such as PCR, 16S rRNA sequencing, loop-mediated isothermal amplification (LAMP), and microarray analysis provide high specificity and sensitivity, yet their high cost, complex sample preparation, and strict laboratory requirements restrict their implementation in routine or resource-limited clinical settings (Hajipour et al., 2021). These limitations support the development of rapid and accessible bacterial recognition strategies that can complement existing diagnostic workflows.

Raman spectroscopy has been increasingly explored for pathogen identification because it can capture molecular-level biochemical information from bacterial cells. Its label-free and non-destructive nature makes it attractive for rapid microbial recognition, but several practical challenges remain before routine clinical translation can be achieved (Tseng et al., 2023). SERS, which relies on metallic nanostructures to amplify Raman signals, improves sensitivity but often suffers from limited reproducibility due to variations in substrate morphology and fabrication stability (Segervald et al., 2025). Batch-to-batch inconsistencies may lead to spectral shifts and signal intensity fluctuations, affecting the reliability of detection. Additionally, localized heating during substrate preparation or measurement may interfere with sensitive biomolecules, potentially compromising spectral stability (Nie et al., 2025). Although confocal Raman microscopy offers high spatial resolution, its high cost, operational complexity, and slow acquisition speed currently limit its suitability for rapid diagnostics or use in primary healthcare settings (Habimana et al., 2020; Pantanella et al., 2013). These issues highlight the need for a more robust, cost-effective, and user-friendly Raman-based approach to enhance its practicality and broaden its applicability in clinical environments.

In the present study, a portable Raman spectrometer was combined with machine learning algorithms for rapid and label-free identification of standardized bacterial suspensions. This work should be regarded as a proof-of-concept study rather than a direct clinical diagnostic test, because all spectra were obtained from prepared bacterial samples after culture, washing, and optical-density adjustment. The portable Raman system has advantages in miniaturization and operational simplicity, allowing spectral data to be acquired within a short time (Sacco Botto et al., 2026). Machine learning helped extract discriminative spectral information from the Raman fingerprints acquired using the portable device, particularly in pairwise classification tasks (Lee et al., 2024). For bacterial pairs with more distinct spectral profiles, such as *P. gingivalis* versus *S. aureus*, the models achieved higher classification accuracy. These differences may be related to variations in cellular biochemical composition, including contributions from cell envelope structures, proteins, lipids, polysaccharides, and pigments, although such assignments should be interpreted as combined spectral patterns rather than single-molecule markers. Regarding the performance of different algorithms in the binary classification tasks, simpler models such as LDA, PA, MLP, and QDA demonstrated robust generalization and acceptable performance. In contrast, high-capacity models and tree-based ensembles, including SVM, ET, GB, and ADB, exhibited obvious signs of overfitting. This discrepancy is fundamentally linked to the high-dimensional characteristics of Raman spectral data, where complex non-linear algorithms are inherently prone to memorizing training noise. Conversely, algorithms that establish simpler or linear decision boundaries act as natural regularizers, enhancing their resistance to overfitting. It is worth noting that all models in this study were rigorously optimized using an automated machine learning framework incorporating Bayesian optimization and cross-validation. Therefore, rather than forcing further artificial parameter tuning—which risks overfitting to the validation set and data leakage—these comparative results inherently reveal that simpler machine learning architectures are more suitable and highly recommended for the robust classification of bacterial Raman spectra (Rahman et al., 2024).

Although the five-class classification accuracy was higher than the balanced random baseline of 20%, the testing accuracy of approximately 50%−60% should still be interpreted cautiously, particularly because the current dataset was generated from pure cultures under standardized sample-preparation conditions. The lower accuracy of the five-class model compared with binary models indicates that simultaneous discrimination of multiple bacterial species remains challenging under the current experimental conditions. This may be attributed to the limited number of independent biological samples, partial overlap of Raman bands among bacterial species, and the restricted spectral resolution and signal-to-noise ratio of the portable Raman instrument. Biological variability and instrumental noise can further increase spectral overlap and class-boundary ambiguity in multiclass classification (Ye et al., 2022). These results also suggest that technical replicate spectra alone cannot fully compensate for limited biological diversity in the dataset, and that spectral features effective for pairwise discrimination may become less robust and more context-dependent as classification complexity increases. Future studies should expand the number and diversity of bacterial isolates, include samples collected across different batches and instruments, and evaluate model performance using external validation datasets, clinical matrices, mixed bacterial communities, and cross-instrument data. More advanced feature extraction approaches, including deep learning models such as convolutional neural networks (CNNs), together with optimized spectral normalization and denoising strategies, may further improve model generalization and multiclass performance (Deng et al., 2022).

One important limitation of this study is the potential confounding effect of culture conditions. The five bacterial species were cultured under conditions suitable for their respective growth requirements, including differences in culture media and oxygen availability. Because Raman spectra are sensitive to bacterial metabolic state and cellular biochemical composition, these culture-related factors may influence spectral profiles (Zhou et al., 2022). Although bacterial cells were washed and resuspended in PBS before Raman acquisition to reduce residual medium interference, this procedure cannot fully remove intracellular metabolic differences or culture-induced changes in the cell envelope (L. Wang et al., 2021). Therefore, the observed classification performance may reflect both species-associated biochemical differences and culture-condition-related physiological variations. Future studies should further assess this issue using harmonized culture conditions where biologically feasible, cross-medium or cross-condition validation, larger independent bacterial collections, and external datasets (Y. Li et al., 2025).

Another limitation of the current workflow is the upstream sample-preparation process. Although Raman spectral acquisition can be completed rapidly, the present protocol requires bacterial cultivation, centrifugation, washing, and OD standardization before measurement. These steps require conventional microbiology laboratory infrastructure and increase the overall turnaround time. Therefore, the current workflow should not be regarded as a complete point-of-care diagnostic platform. Rather, the results demonstrate the feasibility of rapid Raman spectral analysis of prepared bacterial suspensions. From the perspective of pathogen recognition, the main value of this workflow lies in combining intrinsic Raman molecular fingerprints with computational classification, without requiring exogenous labels or species-specific probes. This feature may simplify assay design and reduce reagent dependence, which is relevant to future resource-limited or point-of-care-oriented settings. However, the current results were obtained using standardized bacterial suspensions, and further validation in clinically relevant samples, such as blood, urine, saliva, or mixed microbial communities, is necessary before practical implementation. Future studies should focus on simplifying sample preparation, reducing culture dependence, improving spectral acquisition quality, and validating the approach using clinical specimens and external datasets. Integration of low-cost microscopic Raman systems with optimized machine learning models may also help improve single-cell detection and enhance performance in complex biological matrices (Ho et al., 2019). In addition, because PBS-only blank spectra acquired in the same microcentrifuge tubes were not included in the original design, the Raman band assignments in Table 1 should be interpreted cautiously as putative biochemical contributions. Future studies will include matched blank controls to further improve spectral interpretation.

Furthermore, due to the prohibitive computational cost of performing exhaustive Bayesian hyperparameter optimization (Auto-sklearn) across multiple repeated group-wise data splits, uncertainty estimates such as standard deviations were not calculated in this proof-of-concept study. Future validations utilizing frozen, lightweight models should incorporate bootstrap confidence intervals to provide deeper statistical insights.

In conclusion, portable Raman spectroscopy combined with machine learning provides a feasible label-free workflow for rapid bacterial recognition. The present study demonstrates effective pairwise discrimination among several clinically relevant bacterial species, while also revealing the current limitations of five-class classification using portable Raman data. With larger isolate-level datasets, improved spectral acquisition, and external validation in clinical specimens, this approach may contribute to future development of accessible pathogen identification tools and support more rational antibiotic use.

## 5 Conclusions

In this proof-of-concept study, portable Raman spectroscopy combined with machine learning enabled rapid, label-free recognition of five bacterial species using standardized bacterial suspensions. The workflow showed strong performance in several binary classification tasks, whereas five-class classification remained limited, indicating that simultaneous multiclass recognition is still challenging under the current conditions. Further studies using larger independent bacterial collections, improved preprocessing and acquisition strategies, clinical specimens, external datasets, and cross-instrument validation are needed to improve robustness and generalizability.

## Data Availability

The original contributions presented in the study are included in the article/Supplementary material, further inquiries can be directed to the corresponding authors.
