# Supplementary material for: Portable Raman spectroscopy combined with machine learning for rapid and label-free recognition of five pathogenic bacteria
Source: Front Microbiol. 2026 Aug 6;17:1872435. doi: 10.3389/fmicb.2026.1872435 (PMC13490111; doi:10.3389/fmicb.2026.1872435)
Supplement: Supplementary file 1 [file Table_1.docx]

Supplementary Material

**Portable Raman Spectroscopy Combined with Machine Learning for Rapid and Label-Free Recognition of Five Pathogenic Bacterial Species.**

**Shisheng Cao^1+^, Ran Pang^1,2+^, Yongqiang Chen^1^, Langdi Zhong^1^, Xiaoxi Dong^1^, Hongxiao Li^1*^, Huijuan Yin^1*^**

^1^Institute of Biomedical Engineering, Chinese Academy of Medical Sciences & Peking Union Medical College, Tianjin 300192, China

^2^General Hospital of Tianjin Medical University, Tianjin 300052, China

***Correspondence:** Hongxiao Li ([lihx@bme.pumc.edu.cn](mailto:lihx@bme.pumc.edu.cn)) Huijuan Yin [(yinhj@bme.pumc.edu.cn)](mailto:(yinhj@bme.pumc.edu.cn))

^+^Shisheng Cao and Ran Pang contributed equally to the work and should be regarded as co-first authors.

**Supplementary Table S1. Configuration of the Auto-Sklearn machine learning framework and data partitioning strategy used in this study.**

| **Parameter** | **Setting** |
| --- | --- |
| Auto-Sklearn version | 0.13.0 |
| Reference | Feurer et al., 2019 |
| Candidate algorithms | ADB, ET, GB, LDA, SVM, MLP, PA, QDA |
| include_estimators | One specified learner per training run |
| Hyperparameter search | Default Auto-Sklearn search space for each included learner |
| Optimization metric | Default Auto-Sklearn classification setting |
| Random seed | No custom random seed specified; default Auto-Sklearn setting used |
| time_left_for_this_task | 1,800 s for binary classification; 3,600 s for five-class classification |
| n_jobs | 30 |
| memory_limit | 32,000 MB |
| ensemble_size | 1 |
| Ensemble strategy | Disabled; only the best single model retained |
| resampling_strategy | Cross-validation |
| resampling_strategy_arguments | {'folds': 3} |
| Data split ratio | 75% training/validation; 25% independent test |
| Splitting unit | Physical bacterial sample |
| Data leakage control | All 10 spectra from the same sample assigned to the same partition |

**Supplementary Table S2. Summary of reference strains, physical samples, technical replicate spectra, and sample-level data partitioning for each bacterial species.**

| **Bacterial species** | **Independent isolates/strains** | **Physical samples/culture-derived suspensions** | **Technical replicate spectra per sample** | **Total spectra** | **Training/validation physical samples** | **Training/validation spectra** | **Test physical samples** | **Test spectra** |
| --- | --- | --- | --- | --- | --- | --- | --- | --- |
| *E. coli* | 1 reference strain | 50 | 10 | 500 | 38 | 380 | 12 | 120 |
| *P. aeruginosa* | 1 reference strain | 50 | 10 | 500 | 38 | 380 | 12 | 120 |
| *S. aureus* | 1 reference strain | 50 | 10 | 500 | 38 | 380 | 12 | 120 |
| *P. gingivalis* | 1 reference strain | 50 | 10 | 500 | 38 | 380 | 12 | 120 |
| *S. mutans* | 1 reference strain | 50 | 10 | 500 | 38 | 380 | 12 | 120 |

**Supplementary Table S3. Class-wise precision, recall, F1-score, support, and accuracy of binary classification models for pairwise bacterial discrimination.**

| **Bacterial pair** | **Class** | **Precision** | **Recall** | **F1-score** | **Support** | **Accuracy** |
| --- | --- | --- | --- | --- | --- | --- |
| *E. coli* vs P. aeruginosa | *E. coli* | 0.85 | 0.98 | 0.91 | 119 | 0.91 |
| *E. coli* vs P. aeruginosa | *P. aeruginosa* | 0.98 | 0.83 | 0.9 | 120 | 0.91 |
| *E. coli* vs P. gingivalis | *E. coli* | 0.8 | 0.66 | 0.72 | 119 | 0.74 |
| *E. coli* vs P. gingivalis | *P. gingivalis* | 0.71 | 0.83 | 0.77 | 120 | 0.74 |
| *E. coli* vs S. aureus | *E. coli* | 0.95 | 0.87 | 0.91 | 119 | 0.91 |
| *E. coli* vs S. aureus | *S. aureus* | 0.88 | 0.96 | 0.92 | 120 | 0.91 |
| *E. coli* vs S. mutans | *E. coli* | 0.86 | 0.86 | 0.86 | 119 | 0.86 |
| *E. coli* vs S. mutans | *S. mutans* | 0.86 | 0.87 | 0.86 | 120 | 0.86 |
| *P. aeruginosa* vs P. gingivalis | *P. aeruginosa* | 0.97 | 0.97 | 0.97 | 120 | 0.97 |
| *P. aeruginosa* vs P. gingivalis | *P. gingivalis* | 0.97 | 0.97 | 0.97 | 120 | 0.97 |
| *P. aeruginosa* vs S. aureus | *P. aeruginosa* | 0.97 | 0.72 | 0.83 | 120 | 0.85 |
| *P. aeruginosa* vs S. aureus | *S. aureus* | 0.78 | 0.97 | 0.87 | 120 | 0.85 |
| *P. aeruginosa* vs S. mutans | *P. aeruginosa* | 0.94 | 0.93 | 0.94 | 120 | 0.94 |
| *P. aeruginosa* vs S. mutans | *S. mutans* | 0.93 | 0.94 | 0.94 | 120 | 0.94 |
| *P. gingivalis* vs S. aureus | *P. gingivalis* | 0.97 | 1 | 0.98 | 120 | 0.98 |
| *P. gingivalis* vs S. aureus | *S. aureus* | 1 | 0.97 | 0.98 | 120 | 0.98 |
| *P. gingivalis* vs S. mutans | *P. gingivalis* | 0.76 | 0.96 | 0.85 | 120 | 0.82 |
| *P. gingivalis* vs S. mutans | *S. mutans* | 0.94 | 0.69 | 0.8 | 120 | 0.82 |
| *S. aureus* vs S. mutans | *S. aureus* | 0.82 | 0.78 | 0.8 | 120 | 0.81 |
| *S. aureus* vs S. mutans | *S. mutans* | 0.79 | 0.83 | 0.81 | 120 | 0.81 |

**Supplementary Table S4. MCC and ROC-AUC values of the five-class classification models.**

| **Learner** | **MCC** | **ROC-AUC** |
| --- | --- | --- |
| ADB | 0.207 | 0.689 |
| ET | 0.228 | 0.681 |
| GB | 0.223 | 0.689 |
| LDA | 0.506 | 0.867 |
| SVM | 0.278 | 0.72 |
| MLP | 0.439 | 0.844 |
| PA | 0.452 | 0.841 |
| QDA | 0.377 | 0.774 |

**Supplementary Table S5. Class-wise performance metrics of the five-class classification models**

| **Learner** | **Class** | **Precision** | **Recall** | **F1-score** | **Support** |
| --- | --- | --- | --- | --- | --- |
| ADB | *E. coli* | 0.23 | 0.235 | 0.232 | 119 |
| ADB | *P. aeruginosa* | 0.518 | 0.717 | 0.601 | 120 |
| ADB | *P. gingivalis* | 0.333 | 0.383 | 0.357 | 120 |
| ADB | *S. aureus* | 0.487 | 0.308 | 0.378 | 120 |
| ADB | *S. mutans* | 0.216 | 0.175 | 0.194 | 120 |
| ET | *E. coli* | 0.285 | 0.294 | 0.289 | 119 |
| ET | *P. aeruginosa* | 0.595 | 0.65 | 0.622 | 120 |
| ET | *P. gingivalis* | 0.314 | 0.442 | 0.367 | 120 |
| ET | *S. aureus* | 0.565 | 0.292 | 0.385 | 120 |
| ET | *S. mutans* | 0.237 | 0.225 | 0.231 | 120 |
| GB | *E. coli* | 0.268 | 0.319 | 0.291 | 119 |
| GB | *P. aeruginosa* | 0.686 | 0.6 | 0.64 | 120 |
| GB | *P. gingivalis* | 0.302 | 0.375 | 0.335 | 120 |
| GB | *S. aureus* | 0.511 | 0.383 | 0.438 | 120 |
| GB | *S. mutans* | 0.221 | 0.208 | 0.215 | 120 |
| LDA | *E. coli* | 0.492 | 0.513 | 0.502 | 119 |
| LDA | *P. aeruginosa* | 0.912 | 0.692 | 0.787 | 120 |
| LDA | *P. gingivalis* | 0.497 | 0.733 | 0.593 | 120 |
| LDA | *S. aureus* | 0.722 | 0.542 | 0.619 | 120 |
| LDA | *S. mutans* | 0.538 | 0.525 | 0.532 | 120 |
| SVM | *E. coli* | 0.21 | 0.294 | 0.245 | 119 |
| SVM | *P. aeruginosa* | 0.754 | 0.792 | 0.772 | 120 |
| SVM | *P. gingivalis* | 0.388 | 0.375 | 0.381 | 120 |
| SVM | *S. aureus* | 0.449 | 0.292 | 0.354 | 120 |
| SVM | *S. mutans* | 0.375 | 0.35 | 0.362 | 120 |
| MLP | *E. coli* | 0.435 | 0.42 | 0.427 | 119 |
| MLP | *P. aeruginosa* | 0.783 | 0.692 | 0.735 | 120 |
| MLP | *P. gingivalis* | 0.541 | 0.608 | 0.573 | 120 |
| MLP | *S. aureus* | 0.575 | 0.508 | 0.54 | 120 |
| MLP | *S. mutans* | 0.46 | 0.525 | 0.49 | 120 |
| PA | *E. coli* | 0.431 | 0.706 | 0.535 | 119 |
| PA | *P. aeruginosa* | 0.724 | 0.742 | 0.733 | 120 |
| PA | *P. gingivalis* | 0.566 | 0.358 | 0.439 | 120 |
| PA | *S. aureus* | 0.583 | 0.5 | 0.538 | 120 |
| PA | *S. mutans* | 0.559 | 0.475 | 0.514 | 120 |
| QDA | *E. coli* | 0.454 | 0.538 | 0.492 | 119 |
| QDA | *P. aeruginosa* | 0.842 | 0.708 | 0.769 | 120 |
| QDA | *P. gingivalis* | 0.411 | 0.383 | 0.397 | 120 |
| QDA | *S. aureus* | 0.476 | 0.5 | 0.488 | 120 |
| QDA | *S. mutans* | 0.378 | 0.375 | 0.377 | 120 |
